# FCI: an R-based algorithm for evaluating uncertainty of absolute real-time PCR quantification

**DOI:** 10.1186/1471-2105-9-13

**Published:** 2008-01-10

**Authors:** Paolo Verderio, Sara Pizzamiglio, Fabio Gallo, Simon C Ramsden

**Affiliations:** 1 Unit of Medical Statistics and Biometry, Istituto Nazionale dei Tumori, Milan, Italy; 2 National Genetics Reference Laboratory (Manchester), St. Mary's Hospital, Manchester, UK

## Abstract

**Background:**

FCI is an R code for analyzing data from real-time PCR experiments. This algorithm estimates standard curve features as well as nucleic acid concentrations and confidence intervals according to Fieller's theorem.

**Results:**

In order to describe the features of FCI four situations were selected from real data collected during an international external quality assessment program for quantitative assays based on real-time PCR. The code generates a diagnostic figure suitable for assessing the quality of the quantification process.

**Conclusion:**

We have provided a freeware programme using this algorithm specifically designed to increase the information content of the real-time PCR assay.

## Background

Real-time PCR is widely used for the quantification of nucleic acids in a wide range of clinical and research applications including the measurement of gene dosage, detection of residual disease in haematological malignancies and detection of bacterial and viral infection.

Real-time PCR typically employs fluorescent probes which generate a signal that accumulates during PCR cycling in a manner proportional to the concentration of amplification products. Absolute quantification of a nucleic acid target can be achieved using a standard curve, which is generated by amplifying known amounts of the target DNA. The standard curve is typically generated using a series of 10-fold dilutions of a control template. For each dilution, replicated determinations of the cycle threshold (ct) are performed and a straight line is fitted to the data by plotting the ct averages as a function of the logarithm of the starting concentration of the standards. By applying a technique known as "inverse regression," the straight line is used as a "calibrator" to estimate the unknown starting DNA concentration in the sample under examination.

As in any titration, evaluating the uncertainty in the estimated concentration of the unknown sample is critical for interpreting the data and optimizing experimental procedures. In addition to the point estimate it is important to calculate the confidence interval of the "true" value of the unknown concentration.

Commercial software specifically designed for generating standard curves and estimating nucleic acid concentrations is now available, however, there are currently no freeware tools available for calculating the uncertainty associated with the concentration estimates.

Several approaches have been proposed for constructing confidence intervals in inverse regression [1-5], but the most frequently used method is due to Fieller [6]. Fieller's theorem provides a general procedure for the construction of confidence limits for certain ratios of parameters, most often applied to ratios of linear combinations of parameters (e.g. inverse estimate from a linear regression model). In this note we propose a statistical tool, FCI (Fieller's Confidence Interval), to estimate the confidence interval (CI) of the "true" value of each unknown concentration according to Fieller's theorem.

## Implementation

FCI code has been tested on the WINDOWS platform with the R Software version 2.5.1 [7], an open-source statistical programming language. FCI implementation involves data import, FCI running and FCI output.

**Figure 1:**
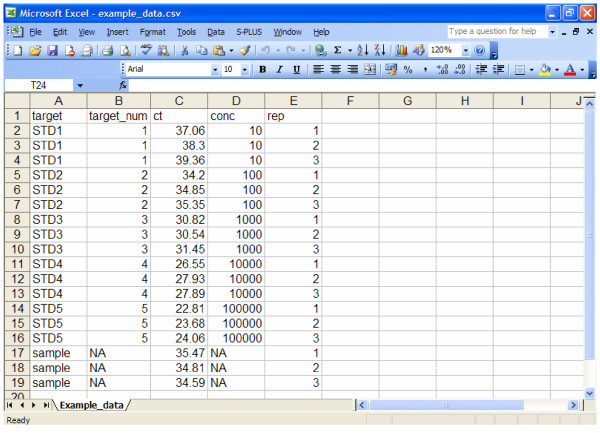
**Screenshot of PCR real time information file in .csv format**. The alphanumeric string "STDi" (with i = 1, 2,...I) identifies each of the I standards and the character string "sample" identifies the unknown sample under investigation. In "target_num" variable, the number i (with i = 1, 2...I) corresponds to each i-th standard and the alphanumeric string "NA" corresponds to the unknown sample. The "ct" variable represents the ct values measured for standards and unknown sample, while the "conc" variable reports the known concentration (copy number) of the standards. Note that, the alphanumeric string NA is inserted in correspondence of the unknown sample. In "rep" variable the number j (with j = 1, 2,...J) identifies each replication for both standards and sample.

Data must be imported into FCI as Comma Separated Values (.csv) file [see Additional File 1] using Microsoft Excel '97 or a more recent version. Figure 1 shows a screenshot of real-time PCR data suitable for analysis by FCI.

Before running the FCI code it is necessary to save the file in the new directory C:\DATA and install the 'car' package from the Packages menu of R.

The FCI algorithm works as follows. First, the data are fitted to a linear regression model as reported in equation 1 and the corresponding analysis of variance table (Anova table, Table 1) is displayed in the output. Next, using the regression coefficient (Intercept and Slope) estimates, the FCI code provides the unknown concentration estimate (equation 2) both in (common) logarithmic scale and also in its original scale as copy number. Finally lower and upper limits of the 100(1-α)% Fieller's CI of the unknown concentration in both logarithmic and original scale are estimated as shown in [8]. By default FCI provides a two-tailed 95% confidence interval (significance level, α = 0.05), however users can modify the confidence level (1-α) by replacing 0.95 in the command line for calling the code with the chosen level.

**Table 1:** Anova Table

**Source**	**df**	**SS**	**MS**	**F.value**
Regression	df_R _= 1	${\text{SS}}_{\text{R}}=\sum_{\text{i}=\text{1}}^{\text{I}}{\text{J}}_{\text{i}}{\left({\hat{\text{y}}}_{\text{i}}-\bar{\bar{\text{y}}}\right)}^{2}$	${\text{MS}}_{\text{R}}=\frac{{\text{SS}}_{\text{R}}}{{\text{df}}_{\text{R}}}$	$\frac{{\text{MS}}_{\text{R}}}{{\text{s}}_{\text{p}}^{\text{2}}}$
Error	${\text{df}}_{\text{E}}=\left(\text{I}-\text{2}\right)+\sum_{\text{i}=\text{1}}^{\text{I}}\left({\text{J}}_{\text{i}}-\text{1}\right)$	${\text{SS}}_{\text{E}}=\sum_{\text{i}=\text{1}}^{\text{I}}\sum_{\text{j}=1}^{{\text{J}}_{\text{i}}}{\left({\text{y}}_{\text{ij}}-{\hat{\text{y}}}_{\text{i}}\right)}^{2}$	${\text{MS}}_{\text{E}}=\frac{{\text{SS}}_{\text{E}}}{{\text{df}}_{\text{E}}}$	
Lack of fit	df_L _= (I – 2)	${\text{SS}}_{\text{L}}=\sum_{\text{i}=\text{1}}^{\text{I}}{\text{J}}_{\text{i}}{\left({\hat{\text{y}}}_{\text{i}}-{\bar{\text{y}}}_{\text{i}}\right)}^{\text{2}}$	${\text{MS}}_{\text{L}}=\frac{{\text{SS}}_{\text{L}}}{{\text{df}}_{\text{L}}}$	$\frac{{\text{MS}}_{\text{L}}}{{\text{s}}_{\text{p}}^{\text{2}}}$
Pure error	${\text{df}}_{\text{P}}=\sum_{\text{i}=\text{1}}^{\text{I}}\left({\text{J}}_{\text{i}}-\text{1}\right)$	${\text{SS}}_{\text{P}}=\sum_{\text{i}=\text{1}}^{\text{I}}\sum_{\text{j}=\text{1}}^{{\text{J}}_{\text{i}}}{\left({\text{y}}_{\text{ij}}-{\bar{\text{y}}}_{\text{i}}\right)}^{\text{2}}$	${\text{s}}_{\text{p}}^{\text{2}}=\frac{{\text{SS}}_{\text{p}}}{{\text{df}}_{\text{p}}}$	
Total corrected	${\text{df}}_{\text{T}}=\left(\sum_{\text{i}=\text{1}}^{\text{I}}{\text{J}}_{\text{i}}\right)-1$	${\text{SS}}_{\text{T}}=\sum_{\text{i}=\text{1}}^{\text{I}}\sum_{\text{j}=\text{1}}^{{\text{J}}_{\text{i}}}{\left({\text{y}}_{\text{ij}}-\bar{\bar{y}}\right)}^{\text{2}}$		

Analysis of variance for the linear regression model underlying the standard curve. In the absence of repeated measurements of ct for all standard dilutions the Error Sum of Squares (SS_E_) can not be broken up into Lack of fit (SS_L_) and Pure error (SS_p_) Sum of Squares. df, degrees of freedom; SS, Sum of Square; MS, Mean Square; ${\hat{\text{y}}}_{\text{i}}$, predicted value corresponding to x_i_; ${\bar{\text{y}}}_{\text{i}}$, mean of the replicated values of ct measured for the i-th standard; $\bar{\bar{\text{y}}}=\sum_{\text{i}=\text{1}}^{\text{I}}\sum_{\text{j}=\text{1}}^{{\text{J}}_{\text{i}}}\left({\text{y}}_{\text{ij}}\right)/\sum_{\text{i}=\text{1}}^{\text{I}}{\text{J}}_{\text{i}}$

The code performs a "Lack of fit" test on the fitted calibration model, the computation of 95% confidence interval for regression coefficients and, where appropriate, provides messages to assist in the interpretation of the results. In addition it generates a diagnostic plot enabling both an assessment of PCR assay quality and also a visual representation of the Fieller's CI graphical derivation [9].

## Results and Discussion

In order to illustrate the functionality of FCI, we considered the data provided by four participants (laboratories A-D) in an international external quality assessment program (EQUAL-quant) for quantitative assays based on real-time PCR [10]. In this program laboratories received primers, labelled probe master mixture, plasmid standards (containing 10, 10^2^, 10^3^, 10^4^, 10^5 ^copies/5 μL), and three samples of unknown concentration (test samples). Participants were required to measure the target copy number in all test samples and provide all ct values in triplicate. For the purposes of this illustration we consider only performance in the analysis of the test sample corresponding to the lowest target copy number (56.21 copies/5 μl calculated as median value of copy number from a total of 92 laboratories).

**Figure 2:**
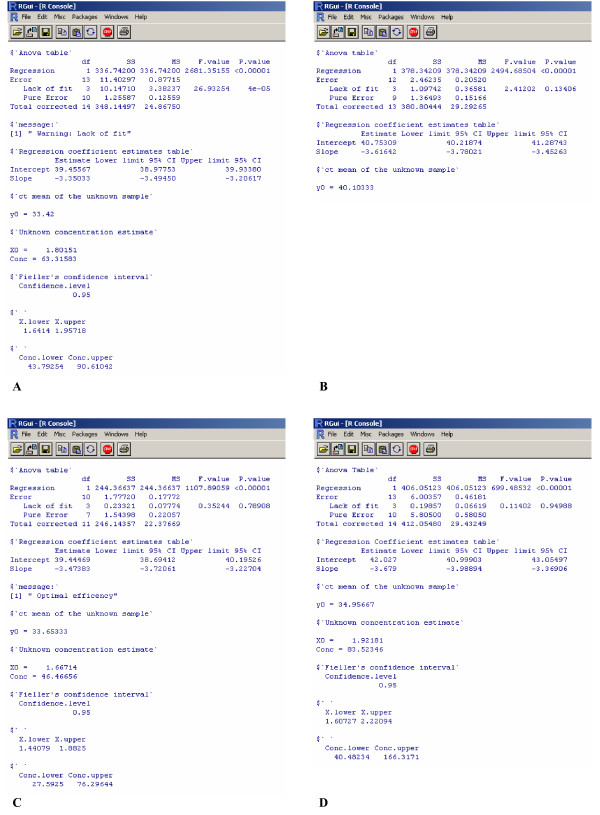
Panel A – D show the FCI output for example A-D, respectively. The FCI output provides the following information: Anova table, summarizes the results of the analysis of variance for the linear regression model underlying the standard curve; Regression Coefficient estimates tables, reports estimates of the standard curve parameters (Intercept and Slope) together with their 95% confidence interval; y0, ct mean of the unknown sample; X0, Unknown concentration estimate in common logarithmic scale; Conc, Unknown concentration estimate in its original scale as copy number; Confidence.level, the chosen confidence level (1-α) of the Fieller's confidence interval; X.lower and X.upper, Lower and upper limits of the 100(1-α)% Fieller's confidence interval of the unknown concentration in logarithmic scale; Conc.lower and Conc.upper Lower and upper limits of the 100(1-α)% Fieller's confidence interval of the unknown concentration in original scale as copy number.

**Figure 3:**
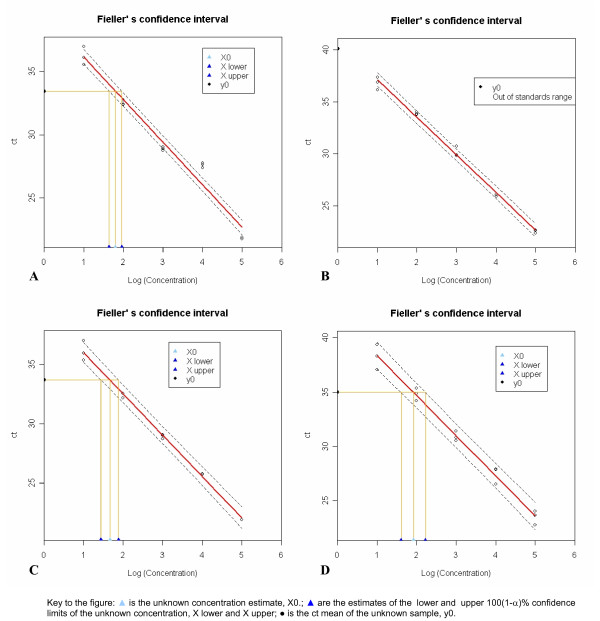
**Fieller's confidence interval**. Panel A-D show the figure provided by FCI for examples A-D, respectively. In each figure the red continuous line is the estimate standard curve and the two black dashed curves are the estimated limits of its 100(1-α)% prediction interval. A horizontal line parallel to the X-axis is drawn at the height of the mean of the ct values (y0) measured in the unknown sample. Where this line intercepts the two dashed curves, as well as the standard curve, three perpendicular lines are traced onto the X-axis giving the lower and the upper 100(1-α)% confidence limits (X.lower and X.upper) of the unknown concentration together with its estimate (X0).

For laboratory A the linear model underlying the standard curve was inadequate. In this case the warning message "Lack of fit" appears in the output (Figure 2, panel A) as the p-value associated to the Lack of fit test is less than 0.05 (significance level α = 0.05). Although FCI code will provide the Fieller's confidence interval, caution should be taken when interpreting results and attempts should be made to discover where and how the inadequacy occurred. In fact, as we see from Figure 3, panel A, the replicates related to standards 4 and 5 appear inadequate to the linear fit.

For laboratory B the ct mean of the unknown sample (y0) lies outside the range of the standard dilutions used to fit the standard curve. In this situation (i.e. extrapolation) we cannot assume that the linear model on which the calibration is based will hold true outside the range of the standard dilutions. In this case the FCI code will not provide the Fieller's CI in the output (Figure 2, panel B), and the warning message "Out of standards range" appears in the legend of the corresponding figure (Figure 3, panel B).

For laboratory C the 95% CI of the true value of the slope includes the theoretical expected slope value (-3.32193) corresponding to 100% amplification efficiency. In such a situation the message "Optimal efficiency" appears in the output (Figure 2, panel C). This message appears only when no warning messages have been displayed. Figure 3, panel C reports the pertinent graph.

Laboratory D illustrates the situation in which no messages, either in the output or in the figure, are displayed (Figure 2, panel D; Figure 3, panel D).

When the 95% confidence interval of the true value of the slope includes zero (an unlikely eventuality in a real-time PCR experiment) the warning message "Not real confidence interval" appears in the FCI output. In this situation, not observed in our real data, the resulting Fieller's confidence interval is infinite in extent and the data have to be considered as valueless for estimating the unknown concentration.

## Conclusion

We have described an algorithm for the computation of CIs based on Fieller's theorem in the context of real-time PCR quantification. While the algorithm is designed in this case for real-time PCR experiments, it is easily adapted to other assays based on inverse estimation from a straight line model (calibration). FCI estimates the standard curve, the unknown sample concentration and its uncertainty. Furthermore it provides an insightful diagnostic figure.

## Methods

The statistical model corresponding to the standard curve is :

(1) y_ij _= β_0 _+ β_1_x_i _+ ε_ij_

where y_ij _specifies the value of ct measured for the j-th replication (j = 1, 2,...,J_i_) at the i-th standard (i = 1, 2,...,I), x_i _defines the logarithm of the starting DNA/cDNA concentration of the i-th standard and ε_ij _is the random component assumed to be normally distributed with mean zero and constant error variance σ^2^. The estimates b_0 _and b_1 _of β_0 _and β_1 _respectively are obtained by the Ordinary Least Squared method.

The uncertainty associated to linear regression model underlying the standard curve can be broken up according to the analysis of variance (Anova) as reported in Table 1[11].

The value of interest in a real-time PCR experiment is the logarithm of the unknown DNA/cDNA concentration in the sample under examination. The latter (x_0_) is usually estimated by resorting to the inverse regression, as:

(2) $${\hat{\text{x}}}_{\text{0}}=\frac{{\bar{\text{y}}}_{\text{0}}-{\text{b}}_{\text{0}}}{{\text{b}}_{\text{1}}}$$

where ${\bar{\text{y}}}_{\text{0}}$ is the mean of the K replicated values of ct [ct_k _= y_k_, (k = 1, 2,....K)] measured for the sample under examination.

As shown in Verderio *et al*. [8] the limits of 100(1-α)% confidence interval of x_0 _according to Fieller's theorem are obtained as roots of a second degree equation.

## Availability and requirements

For the current version of FCI code please see Additional file 2. The commands for importing data, running the code and displaying the output are provided in Additional file 3. Further development as well as extended version of FCI code will be available trough our website: . FCI code use R Software , an open-source statistical programming language.

## Authors' contributions

All authors contributed to the work described in the manuscript. The R-implementation of the FCI code as well as its testing was carried out by FG with advice and supervisor from PV and SP. PV provided oversight of the work, finalized the draft and optimized the final version. SR supplied the data for testing the code and improved the writing. All authors read and approved the manuscript.

## Supplementary Material

Additional file 1 A .csv file containing the data of example D on which are based the output in figure 2, panel D and the graph in figure 3, panel D. Click here for file

Additional file 2 A text file containing the FCI code written in R language. Click here for file

Additional file 3 A text file containing the commands for importing data, calling the code and displaying the output. Click here for file
